# Beneficial effects of benzodiazepine on masticatory muscle dysfunction induced by chronic stress and occlusal instability in an experimental animal study

**DOI:** 10.1038/s41598-020-65524-w

**Published:** 2020-05-29

**Authors:** Glauce C. Nascimento, Bruno L. Malzone, Daniela M. Iyomasa, Yamba C. L. Pereira, João Paulo M. Issa, Christie R. A. Leite-Panissi, Ii-Sei Watanabe, Mamie M. Iyomasa, Ramon Fuentes, Elaine Del Bel, Fernando J. Dias

**Affiliations:** 1 Department of Basic and Oral Biology, School of Dentistry of Ribeirão Preto, Universidade de São Paulo, Ribeirão Preto, SP Brazil; 2 Department of Morphology, Presidente Prudente Medical School, Universidade do Oeste Paulista, Presidente Prudente, SP Brazil; 3 Department of Anatomy, Institute of Biomedical Sciences, Universidade de São Paulo, São Paulo, SP Brazil; 4 Department of Integral Dentistry, Research Centre for Dental Sciences (CICO), Dental School, Universidad de La Frontera, Temuco, Chile

**Keywords:** Human behaviour, Neurological disorders

## Abstract

Psychological stress and occlusal alteration are important etiologic factors for temporomandibular/masticatory muscular disorders. In particular, the exact physiologic mechanism underlying the relation by occlusal alteration and temporomandibular disorders remains unclear. Our purpose was to test the hypothesis that benzodiazepine therapy is able to prevent metabolic and vascular changes in the medial pterygoid muscle of rats under chronic stress after 14 days of unilateral exodontia. Adult Wistar rats were submitted to unpredictable chronic mild stress (10 days) and/or unilateral exodontia and their plasma and medial pterygoid muscles were removed for analysis. A pre-treatment with diazepam was used to verify its effect on stress. The parameters evaluated included anxiety behavior, plasma levels of corticosterone, metabolic activity by succinate dehydrogenase, capillary density by laminin staining and ultrastructural findings by transmission electron microscopy. Occlusal instability induced anxiety-like behavior on elevated plus-maze test and diazepam administration blocked the appearance of this behavior. Unilateral exodontia promoted in the contralateral muscle an increase of oxidative fibers and capillaries and modification of sarcoplasmic reticulum. Chronic stress caused increased glycolytic metabolism, reduced capillary density and morphological changes in mitochondria on both sides. Association of both factors induced a glycolytic pattern in muscle and hemodynamic changes. Pharmacological manipulation with diazepam inhibited the changes in the medial pterygoid muscle after stress. Our results reveal a preventive benzodiazepine treatment for stress and occlusal instability conditions affecting masticatory muscle disorders. In addition, provide insights into the mechanisms by which chronic stress and exodontia might be involved in the pathophysiology of masticatory muscular dysfunctions.

## Introduction

Temporomandibular dysfunction (TMD) can present itself as muscular, articular, or of dual character, when it affects both systems^1^. Particularly, many findings suggested these dysfunctions are associated with abnormal physiology of the masticatory muscle and consider this an important risk indicator for TMD development^2^. In addition, their risk factors encompass structural, physiological, behavioral, and environmental characteristics. Association between emotional stress and occlusal instability, in case of orthodontic treatment, masticatory disorders and the removal of third molars, have been involved with the development of temporomandibular joint disorders and maintenance of pain^3^.

Regarding occlusal interferences, they can be associated with fatigue and masticatory muscle pain^4^. In this context, the adaptation of these muscles has been analyzed after exodontia^5,6^ and other occlusal interferences in experimental models. Bazan *et al*.^5^ found that unilateral exodontia in guinea pigs decreases metabolic activity of the medial pterygoid muscle (MPM) in the same side due to prolonged hypofunction. In addition, the influence of occlusal alteration in the MPM of gerbils, previously described by Iyomasa *et al*.^7^, shows muscle fibers with a central nucleus and decreased diameter six days after unilateral exodontia. It has been admitted, therefore, that dysfunctional occlusion can affect morphology and function of masticatory muscles^8,9^.

TMD is related with anxiety, stress and depression^9,10^. For this reason, the involvement of this disorder with emotional disturbances arouses the interest of researchers and stimulates basic and clinical research in the area. In accordance, emotional stress alters oral functioning^11^ and sensory perception^12^. Also, stress-induced muscle disorders cause pain^13^ through mechanisms that involve modifications in muscle metabolism, homeostatic balance and inflammatory processes^14^. However, while mandibular elevator muscles are intensively affected by stress^15^, the mechanism by which stress can alter orofacial muscular physiology has not yet been elucidated. Pre-clinical investigations have invested in animal models to study stress- related disorders and these models have shown effectiveness to answer questions inherent to the relationship between stress and orofacial muscle changes. Particularly, unpredictable chronic mild stress (UCMS) protocol is a valid animal model used to investigate functional and behavioral alterations produced by chronic stress^16^. Briefly, in this test, rodents are exposed to a variety of relatively mild stressors intermittently, generally for two to four weeks^17^.

**Figure 1:**
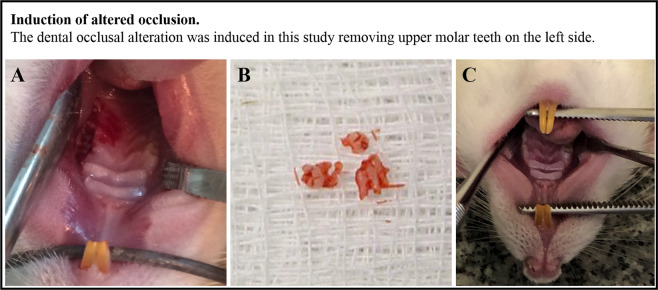
Representative images from rats immediately after the unilateral exodontia (**A**); extracted molar teeth (**B**) and alveolus of rats 14 days after unilateral extraction (**C**).

This study hypothesizes that unpredictable chronic mild stress influences on morphophysiological changes in the right and left MPMs induced by occlusal instability (Fig. 1) and that classic pharmacological manipulation with benzodiazepine reverses the alterations caused, since diazepam shows anti-stressor and anxiolytic-like activities. The aim of this work is to contribute to proposing a new therapeutic targ*et al*ternative for orofacial muscular dysfunctions and the emotional risk factors associated. In addition, this study aims to propose mechanisms by which stress acts to modify metabolism and capillary patterns of the MPM.

## Results

### Anxiety-like behavior

**Figure 2:**
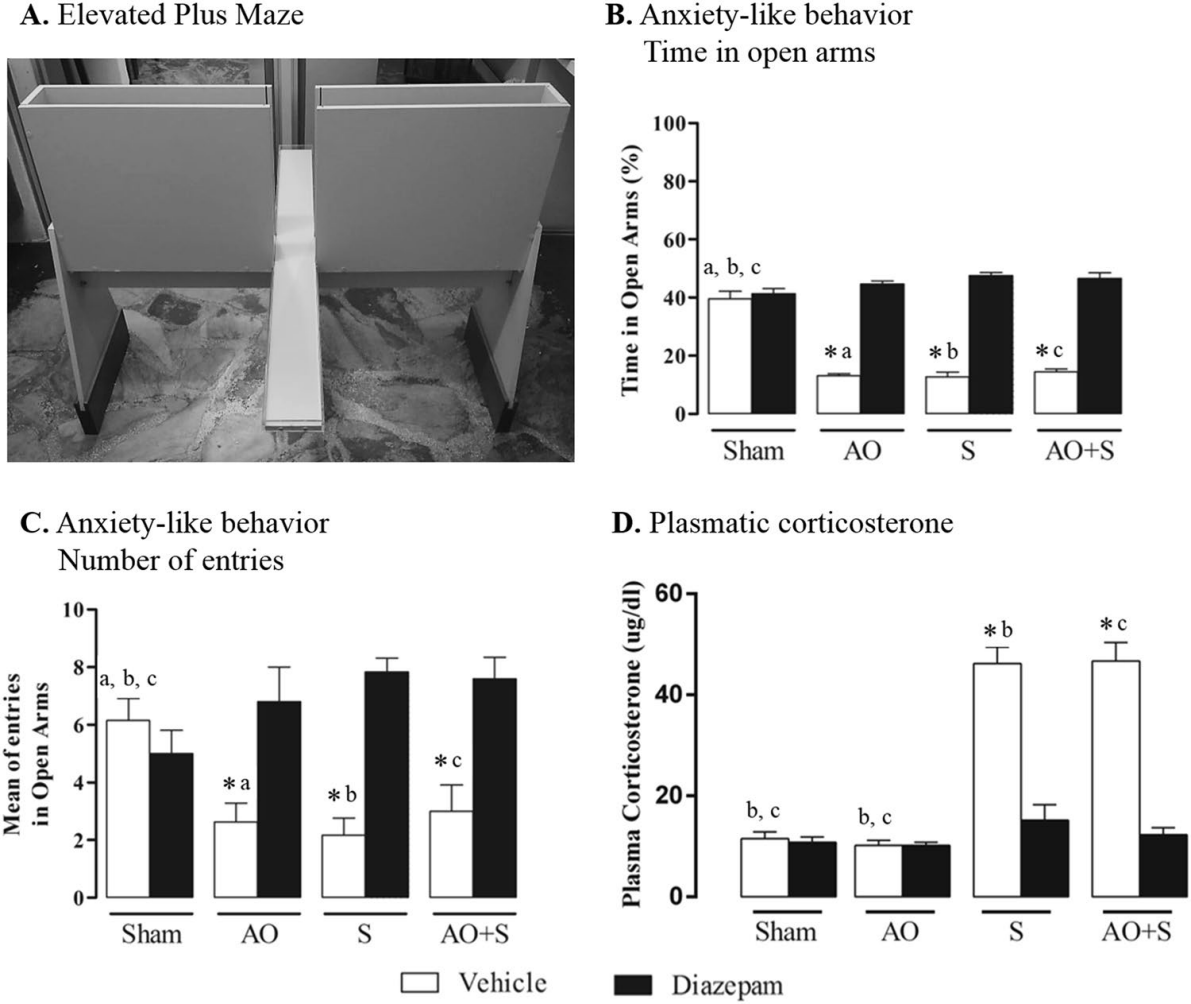
Effects of stress, occlusal instability and diazepam treatment on anxiety-like behavior and corticosterone hormone. (**A**) Graphical representation from EPM apparatus. Effects of stress and unilateral exodontia on time spent in open arms (**B**) and on the percentage of entries into the open arms (**C**) in the EPM test. Two-way ANOVA showed an effect of diazepam therapy in rats submitted to altered occlusion (AO), chronic stress (S) or both (AO + S) (n = 10 per group). (**D**) Analysis of plasma corticosterone levels of control rats and rats submitted to altered occlusion (AO), chronic stress (S) or both (AO + S) (n = 8 per group). Two-way ANOVA showed an effect of chronic unpredictable stress, in which S and AO + S groups presented increased plasma corticosterone compared to the other groups. Data are expressed as the means ± SEM. *P < 0.05 vs diazepam treatment; ^a^P < 0.05 vs AO + vehicle; ^b^P < 0.05 vs S + vehicle; ^c^P < 0.05 vs AO + S + vehicle. SEM: standard error means.

A reduction in open arms entries is an indicative of an anxiolytic-like behavior. Considering the percentage of time spent in open arms, the application of a two-way ANOVA showed a significant decrease (Newman Keuls test) in time spent by rats that underwent molar exodontia and/or stress treated with vehicle compared to that one treated with diazepam (*P* < 0.05; Fig. 2B). Regarding mean entries into open arms (Fig. 2B), we also observed that benzodiazepine treatment is effective in preventing anxiolytic effect for both conditions (*P* < 0.05; Fig. 2C).

### Plasma corticosterone levels

The plasma corticosterone concentrations in experimental groups are shown in Fig. 2D. The chronic unpredictable stress groups revealed a significant increase (*P* < 0.05) in plasma concentrations of corticosterone compared to the other experimental groups. Diazepam administration reverted this state in stressed rats.

### Metabolic activity of muscle fibers

**Figure 3:**
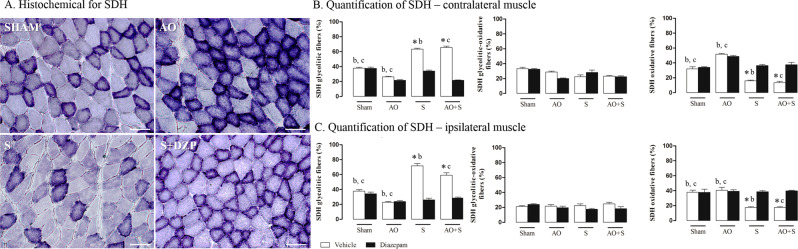
Effects of stress, occlusal instability and diazepam treatment on succinate dehydrogenase enzyme (SDH). (**A**) Panels show representative photomicrographs of SDH staining of the right MPM of control rats (SHAM) and rats submitted to Altered Occlusion (AO), chronic stress (S) or stressed group treated with diazepam (S + DZP), 40× magnification. (**B**) Demonstration of SDH in different groups of rats. Graph indicates number of glycolytic fibers (light), glycolytic-oxidative fibers (intermediate) and oxidative fibers (dark) in control rats (Sham) and rats submitted to altered occlusion (AO), chronic stress (S) or both (AO + S) (n = 8 per group) in contralateral and ipsilateral muscles. Two-way ANOVA indicated an effect of exodontia and stress, isolated or associated. Data were expressed as mean ± SEM. SEM: standard error means. *P < 0.05 vs diazepam treatment; ^b^P < 0.05 vs S + vehicle; ^c^P < 0.05 vs AO + S + vehicle. SEM: standard error means.

The representative photomicrographs from experimental groups are presented in Fig. 3A. Quantitative analysis of this reaction may be seen in Fig. 3B,C. About the contralateral muscle to the exodontia, subjects under stress protocol (S and AO + S groups) treated with vehicle, presented increased glycolitic fibers when compared to the same groups treated with diazepam (*P* < 0.05; Fig. 3B). Moreover, the same stressed rats (S and AO + S groups) treated with vehicle have reduced oxidative fibers in the contralateral muscle when compared to the same groups treated with diazepam (*P* < 0.05; Fig. 3B). Altered occlusion groups presented decreased glycolytic and increased oxidative fibers compared to Sham groups (*P* < 0.05; Fig. 3B). Regarding ipsilateral muscle to the exodontia, Groups of rats submitted to stress protocol (S and AO + S groups) and treated with vehicle of diazepam, present increased glycolytic fibers and decreased oxidative fibers when compared to the same groups treated with diazepam (*P* < 0.05; Fig. 3C). Altered occlusion groups presented decreased glycolytic fibers compared to Sham groups (*P* < 0.05; Fig. 3C).

### Capillary assessment

**Figure 4:**
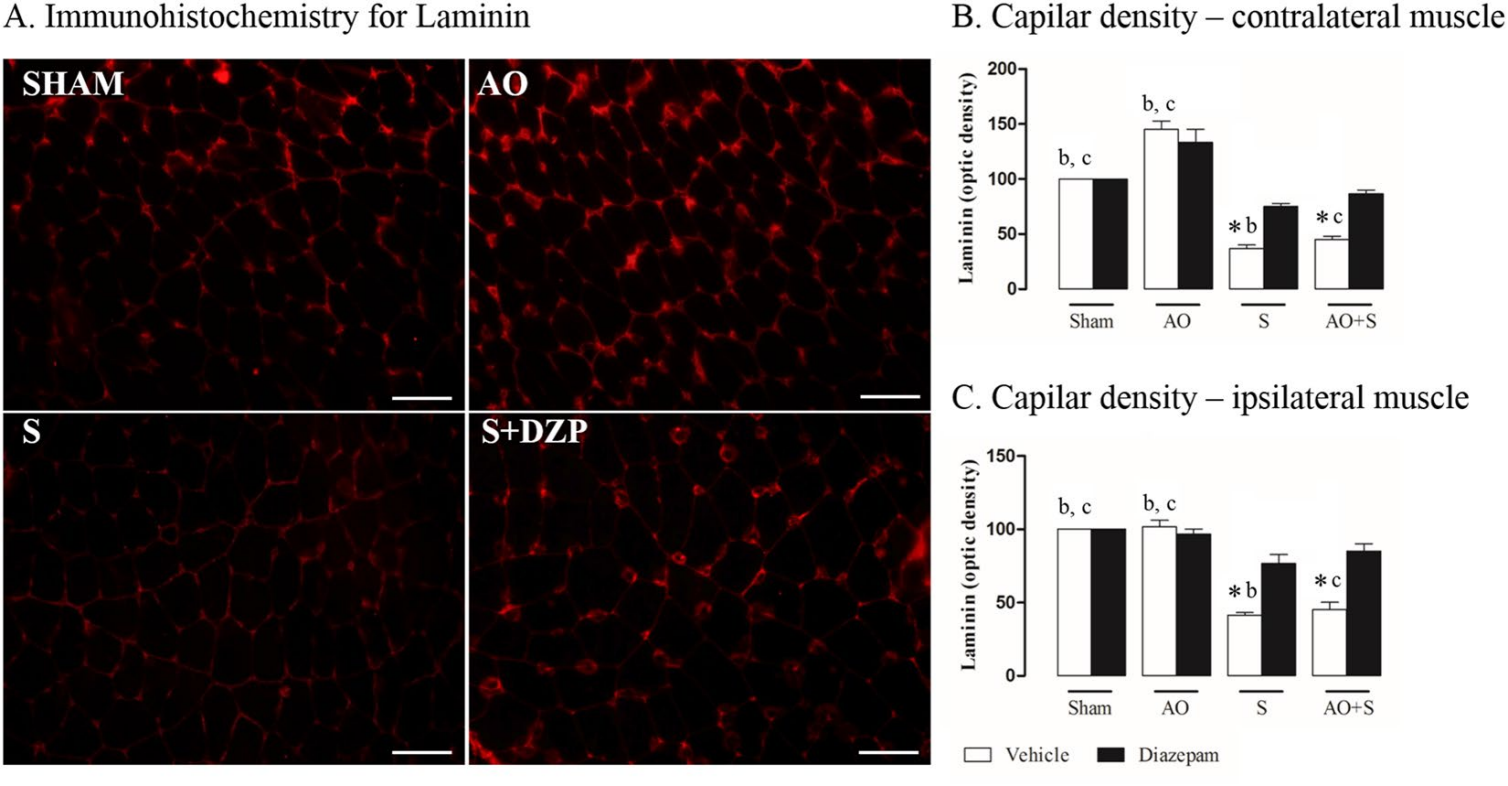
Effects of stress, occlusal instability and diazepam treatment on muscle capillary pattern. (**A**) Panels show representative photomicrographs of immunofluorescence for capillary marked by laminin in the right MPM of control rats (SHAM) and rats submitted to Altered Occlusion (AO), chronic stress (S) or stressed group treated with diazepam (S + DZP), 40× magnification. (**B**) Graphs indicate number of capillaries marked by laminin in control rats (Sham) and rats submitted to altered occlusion (AO), chronic stress (S) or both (AO + S) (n = 8 per group) in contralateral and ipsilateral muscles. Two-way ANOVA indicated an effect of stress and exodontia, isolated or associated. Data were expressed as mean ± SEM. SEM: standard error means. *P < 0.05 vs diazepam treatment; ^b^P < 0.05 vs S + vehicle; ^c^P < 0.05 vs AO + S + vehicle. SEM: standard error means.

For analysis of local vascularization, we quantified laminin in the MPMs (Fig. 4). Representative photomicrographs from the experimental groups are presented in Fig. 4A. Graphs present quantitative analysis from this reaction (Fig. 4B,C). About contralateral muscle to exodontia, rats with altered occlusion (AO group), treated or not with diazepam, present increased laminin when compared to all other experimental groups (*P* < 0.05; Fig. 4B). Groups under stress protocol (S and AO + S groups) treated with vehicle of diazepam, show decreased laminin compared to the same groups treated with diazepam (*P* < 0.05; Fig. 4B). The ipsilateral muscle of rats submitted to stress protocol (S and AO + S groups) and treated with vehicle, showed decreased laminin compared to the same groups treated with diazepam (*P* < 0.05; Fig. 4C).

### Ultrastructural analysis

**Figure 5:**
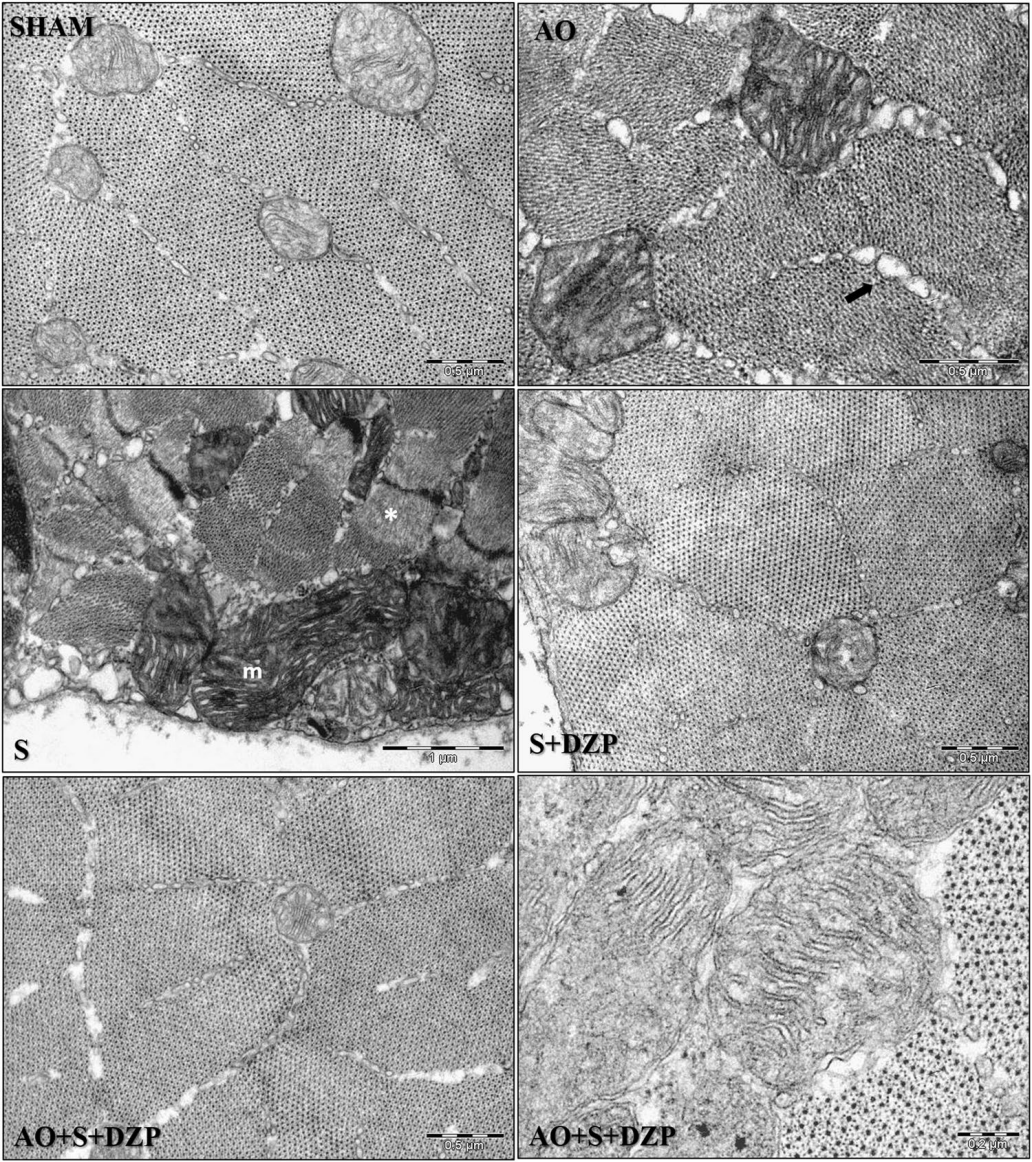
Effects of stress, occlusal instability and diazepam treatment on ultrastructural morphology of medial pterygoid muscle. Transmission Electron Microscopy (TEM) images of right MPM of control animals (SHAM), Altered Occlusion rats (AO), chronic stress group(S), stressed group treated with diazepam (S + DZP) and Altered Occlusion and stress treated with diazepam (AO + S + DZP). AO group showed dilated sarcoplasmic reticulum cisternae with irregular contours (arrow). The S group indicated myofibrils containing amorphous material (*) and altered morphology mitochondria (m). SHAM, S + DZP and AO + S + DZP groups showed muscle fibers with well- preserved structures, such as myofibrils, mitochondrial shape and content, and sarcoplasmic reticulum cisternae without apparent expansion. Scale bars represent: SHAM (0.5 µm), AO (0.5 µm), S (1 µm), S + DZP (0.5 µm), AO + S + DZP (0.5 µm) and AO + S + DZP (2 µm).

Under transmission electron microscopy (Fig. 5), medial pterygoid muscle of control animals (sham group) showed muscle fibers with several well-preserved structures. Myofibrils (myosin and actin) were observed without changes evenly distributed in the cross-sectional area, in addition to well-preserved mitochondria with dilated double membrane interspace and space between mitochondrial cristae, and cisterns of the sarcoplasmic reticulum without apparent dilation around the muscle fibers. Animals in groups AO and S showed different characteristics from those observed in control animals.

The rats submitted to unilateral exodontia (Altered Occlusion) showed dilated sarcoplasmatic reticulum and T-tubules. There were numerous smoothly contoured mitochondria, with ample inter-membrane spaces and dilated space between mitochondrial cristae. The main characteristics of the stressed group (Stress) included muscular fiber cytoplasm – made up of myofibrils surrounded by several vesicles of sarcoplasmic reticulum – and sarcolemma folds that were poorly delineated with irregular contours, some filled with amorphous substance. Additionally, there were disorganized mitochondrial cristae and subsarcolemmal mitochondria with varied sizes.

The animals submitted to malocclusion and the stress protocol that received diazepam pre-treatment (S + DZP and AO + S + DZP groups) presented ultrastructural characteristics similar to animals in the sham group, with a more uniform distribution of myofibrils, more regular and less electrondense mitochondria content compared to animals in groups AO and S, in addition to cisterns of the sarcoplasmic reticulum apparently not dilated.

## Discussion

The present study follows a model of dental occlusion alteration used in our previous studies with effective results^6,7,17^. Once more, the unpredictable chronic stress protocol used herein constituted a valorous tool, providing a realistic model for the stresses of daily life^17,18^. This protocol is closer to reality when compared with repeated chronic stress, since stress situations are not constant. About acute stress, this is a protocol that reproduces a single and punctual stressful factor, what could limit the study^16^. Importantly, the present study differs from the studies cited previously, as it was a pioneer in elucidating metabolic and vascular structural changes prevented by the use of benzodiazepine in medial pterygoid muscle of rats subjected to unpredictable chronic stress.

Our result suggests a strong relationship between occlusal instabilities and anxiolytic behavior. The anxiety-like behavior analyzed here via EPM test presents an important result regarding the ability of the altered occlusion experimental model to induce anxiety behaviors in the same proportion as stress, represented by the reduction of rats entering open arms of the apparatus. This suggestion corroborates findings about the relationship between somatosensory amplification, anxiety behavior and oral behaviors in individuals with facial TMD pain^19^. The relation between anxiety and oral behaviors has been largely verified. Anxious people have frequent oral behaviors episodes as well as high levels of anxiety are also a characteristic of individuals with facial pain^19^.

At note, somatosensory amplification means the tendency to perceive a normal somatic sensation as intense and noxious. In this sense, oral behaviors as repetitive tooth-to-tooth contact and clenching may be more prevalent in individuals with greater somatosensory amplification. In this context, it is reasonable to links painful temporomandibular disorders to increased occlusal awareness that can be explained by the stronger relationship between somatosensory amplification and oral behaviors in individuals with facial TMD pain^19^.

Considering that diazepam was effective not only in groups of animals submitted to the stress protocol, but also on occlusal instability, an important point is about the validation of this type of occlusal alteration as an experimental model that induces an anxiogenic condition. The present work investigated also plasma corticosterone levels in the different experimental groups in order to measure the effectiveness of this protocol, evidencing that the group of animals treated with diazepam vehicle and submitted to the stress protocol alone or with exodontia presented significant increase of corticosterone when compared to the other groups under analysis. Again, diazepam was effective in preventing this increase, suggesting its efficacy on emotional alterations.

The MPM is an even muscle and one of the four main muscles responsible for the chewing function. It has an important relationship with the temporomandibular joint, as a potent masticatory muscle involved in mandibular elevation and eccentric movements of protrusion and laterality in humans^20^. The analysis of a muscle that is involved in all these actions will provide more complete information about possible changes associated with non-physiological muscular activity^21^.In rodents, the MPM (or internal pterygoid) acts mainly by elevating the mandible in conjunction with the masseter and temporal muscles. However, this muscle represents only 12% of the volume of all masticatory muscles, thus being the smallest of the three involved in mandibular elevation^22,23^. Thus, modifications resulting from changes in masticatory function could be more easily evident in this muscle.

With respect to the types of skeletal muscle fibers, these have been classified based on their contraction rate and type of metabolism recruited. They include slow- contracting oxidative fibers; fast-contracting glycolytic-oxidative fibers; and fast- contracting glycolytic fibers^24^. Exodontia in this study (AO group) promoted an increase of oxidative fibers in the right MPM, which use oxidative phosphorylation for metabolism. These fibers have a high amount of myoglobin as an oxygen transport molecule, which presents pigmentation that leaves the fiber darker in relation to glycolytic fibers, which are clear^25^. This increase of oxidative metabolism after exodontia, together with an increase in local vascularization, allows greater diffusion of oxygen to the mitochondria. These results suggest, therefore, that the muscle will take longer to enter into fatigue^26^. Therefore, modified occlusion of the left side, from onset, promotes a protection mechanism in the contralateral muscle, since it will have increased function to compensate the affected side.

In addition, our results show that the induction of chronic stress alters the protective character of the contralateral muscle promoted by exodontia, since it entails an increase in glycolytic metabolism, which depends on anaerobic glycolysis for the synthesis of ATP, and therefore, the muscle tends to enter into fatigue more quickly^26,27^. In addition, we found that there was a reduction of local vascularization and altered morphology of mitochondria, suggesting a decrease in energy for this muscle’s fibers. The stress stimulus alone, in turn, also promotes increased glycolytic metabolism, reduced vascularity and mitochondrial morphological alteration in both sides. Furthermore, glycolytic fibers have a larger diameter than oxidative fibers. The combination of larger size, smaller quantity of myoglobin and fewer blood vessels makes it more likely that glycolytic fibers will run out of oxygen after repeated contractions. Diazepam, in this context, was able to prevent the state produced by isolated stress or by the association of stress and exodontia, as it maintains the oxidative capacity of muscular fibers at normal levels.

Regarding muscular tissue and ultrastructural modifications, the results of this study for the groups administered with diazepam vehicle indicated that the unilateral exodontia (AO group) promoted an increase of oxidative fibers in the contralateral side, increase of capillaries evidenced by laminin, at the ultrastructural level, changes in the arrangement of microfibrils were observed, with no shape and mitochondrial content and sarcoplasmic reticulum with dilated cisterns in the medial pterygoid muscle fibers. Stress alone (S group), on the other hand, caused an increase in glycolytic fibers, a decrease of laminin, and changes in shape and mitochondrial content, in addition to also presenting dilated sarcoplasmic reticulum cisterns and altered myofibril distribution in the muscles studied on both sides. The association of exodontia and stress, in turn, promoted an increase of glycolytic fibers, reduction of laminin and mitochondria in the muscles contralateral to the exodontia. In the animals pharmacologically manipulated with diazepam (AO + DZP and AO + S + DZP groups), we noted an inhibition of changes in the medial pterygoid muscle after stress, which presented similar characteristics to the control group. At the ultrastructural level, the distribution of myofibrils, the mitochondrial shape and content and the sarcoplasmic reticulum did not present any alterations, thus being similar to the characteristics observed in sham animals.

These data indicate that benzodiazepine action was specific to the stress used and did not act as a muscle relaxant, since only the groups with the stress protocol showed a reversal of the altered muscle states. Also, the preventive character of this drug on MPM is emphasized, considering that the treatment was performed 30 minutes before daily stresses. Despite the scarcity of data on the performance of benzodiazepines in muscle disorders associated with TMDs, this study suggests this pharmacological therapy as a positive strategy for clinical conditions involving stress, masticatory muscle disorders and dental occlusal changes. Muscle relaxants of central action minimize skeletal muscle tone and, thus, may promote prevention and reduction of muscle activity associated to TMD of muscular origin. The benzodiazepines act on GABA receptor system that modulates inhibitory synaptic connection throughout the Central Nervous System (CNS), what explain the relaxant effect. Analgesic compounds targeting GABA transporters or GABA related enzymes and receptors are also being developed. In this sense, part of GABA analogs inhibits ion channels as an analgesic mechanism. Nociceptive nerve endings of muscles are rich in receptor molecules for endogenous pain-producing and agents that produce peripheric or central sensitization. The purinergic receptors, for example, can be activated by adenosine triphosphate (ATP), that is released from the muscle by mechanical stimulation of the muscle or contraction^28^. In addition, vanilloid receptors are sensitive to protons (reduced pH) and are also present in muscle nerve endings.

Despite the positive findings about these drugs, the use of benzodiazepines for TMD must be careful due adverse drug responses. In this way, drowsiness, impaired coordination, confusion, amnesia, tolerance and physical dependence can develop in a prolonged period of use of these medications.

This work highlights the preventive effectiveness of a benzodiazepine drug on masticatory muscular dysfunction changes induced by an unpredictable chronic stress experimental model. Furthermore, the data from this study suggest the change of respiratory metabolism and capillary alterations as possible mechanisms involved in the emergence of orofacial muscular dysfunctions in conditions of emotional disorder. These emotional disorders deserve special mention in this study, since, for the first time, there is evidence for anxious behavior induced by an experimental model of occlusal instability. The pathway by which stress promotes muscular metabolic pattern modification remains an important topic for further research.

## Methods

### Animals

A case-control experimental animal study was conducted in accordance with the Animal Care and Use program of the National Institutes of Health (NIH) at United States of America. The experimental protocol was approved by the Animal Use and Ethics Committee of Ribeirao Preto – University of Sao Paulo (Certificate Number: 12.1.418.53.0) and all efforts were made to decrease the number of rats used and to minimize animal suffering. A total of 128 male Wistar rats, weighing 275 ± 25 g, were obtained from Ribeirão Preto Campus, at Universidade de São Paulo, Brazil. The animals were kept in a temperature of 24 ± 1 °C and are submitted to 12 h light/dark cycles, with water and food *ad libitum*.

The animals were distributed in the following eight groups (n = 8 for each group):

- Sham + Vehicle: rats were submitted to simulation of exodontia; they were not exposed to stress protocol and they were administered with vehicle of diazepam;
- Sham + Diazepam: rats were submitted to simulation of exodontia; they were not exposed to stress protocol and they were administered with diazepam;
- AO + Vehicle: Altered occlusion, rats were submitted to unilateral exodontia, they were not exposed to stress protocol and they were administered with vehicle of diazepam;
- AO + Diazepam: Altered occlusion, rats were submitted to unilateral exodontia, they were not exposed to stress protocol and they were administered with diazepam;
- S + Vehicle: Stress group, rats were submitted to simulation of exodontia, they were submitted to unpredictable chronic mild stress protocol and they were administered with vehicle of diazepam;
- S + Diazepam: Stress group, rats were submitted to simulation of exodontia, they were submitted to unpredictable chronic mild stress protocol and they were administered with diazepam;
- AO + S + Vehicle: rats were submitted to unilateral exodontia and unpredictable chronic mild stress protocol and they were administered with vehicle of diazepam;
- AO + S + Vehicle: rats were submitted to unilateral exodontia and unpredictable chronic mild stress protocol and they were administered with diazepam.

### Anxiolytic drug administration

Anxiolytic diazepam (2 mg/kg) was administrated to the animals 30 minutes before the stress protocols, for all 10 days of stress protocol via gavage^29^. The vehicle was prepared with 2% polyethylene sorbitan monooleate (tween 80) in saline solution and administered 200 μl in each animal under the same conditions as diazepam. The drug was administered once a day and in the groups without stress protocol, we started the administration at 14 day of the beginning of the experiments.

### Induction of altered occlusion

The dental occlusal alteration was induced in this study removing upper molar teeth on the left side. The sham groups were subjected to the same trauma by a simulation of jaw opening for teeth extraction. These rats were anesthetized and received the same dose of antibiotic and anti-inflammatory treatments. Their mouths were also opened with an instrument, but we did not perform any incision. The animals were administrated with intraperitoneal anesthesia— tribromoethanol (0.25 g/kg of body weight). We also administered a single dose of antibiotic (Pentabiotic – Zoetis Ltda – Uso veterinário – 3057 H) – penicillin 24,000 IU/kg of body weight, and anti-inflammatory and analgesic (Bananine - flumexine meglumine - Schering-Plorigh, 25 mg/kg, 10 mg/ml) as a prophylactic conduct. After 14 days of the surgery, there is a complete healing of the operatory area. In addition, the socket is occupied by reticular bone and there is an intense bone activity in the trabecular surfaces^30^.

### Chronic unpredictable stress protocol

After surgical recuperation from exodontia procedure (14 days), UCMS protocol was applied in animals from the stress groups (S and AO + S groups). This protocol was performed according to a previous study by our research group^17^. During a ten-day period (14^th^ to 23^rd^ day), five different methodologies were performed, according to the following Table 1:

**Table 1:** 10-day representative schedule of stressor agents used during the experiment.

Day of experiment	Stressor used	Time
1^st^ and 6^th^ days	Agitation (Rats were individually placed in a box on a shaker table with a velocity of 50 rpm)	15 minutes
2^nd^ and 7^th^ days	Forced Swim (Rats swam in a circular container with water and a diameter of 47 cm and depth of 54 cm)	15 minutes
3^rd^ and 8^th^ days	Physical Restraint (Rats were placed in a metal box, which restricted their movements, with 15 cm long and 5 cm in diameter, with adequate ventilation)	2 hours
4^th^ and 9^th^ days	Cold stress (Rats placed in individual boxes were exposed to 4 ^o^C)	30 minutes
5^th^ and 10^th^ days	Deprivation of water (Water was private from rats)	24 hours

Veterinary and technical staff compounds a laboratory technical team that helps in the maintenance of animal health and welfare. The animals were also monitored daily to checking animal activity and self- cleaning, appearance, vocalization and weight monitoring, which is controlled each 3 days. There were no deaths correlated to the stress protocol.

### Evaluation of anxiety-like behavior with the Elevated Plus-maze (EPM) test

The anxiety test was performed one day after the final of stress protocol and plasma was collected one day after this behavioral test. The elevated plus-maze test (EPM) has been proven to be bi-directionally sensitive to detection of anxiety in rats. This test includes approach-avoidance paradigms representing the conflict between rat’s innate exploratory activity and the behavior to avoid light, open and elevated spaces. The EPM was constructed according to the specifications determined by Morato and Brandao^31^. The rat´s behavior in the EPM was recorded by a system of camera linked to a computer, situated outside the experimental room. Rats were placed individually in the center of the EPM facing the side of the closed arms. At the end of each session, rats were returned to the home cages, and the maze was cleaned with alcohol solution (70%). The number of entries and the time spent into the open arms of the maze were analyzed during a 5-min test time. The time spent in the open arms is given by percentage in a total time of 300 seconds spent in the maze. The Geo Vision software was used to behavioral scores.

### Euthanasia and tissue processing

The eight groups of rats were experimented repeated twice (n = 64 each time) for the acquisition of tissue in two manners: one experiment was used for immunohistochemical technique and the other one for the rest of the tests.

For immunocytochemistry, one day after the behavioral analysis, it was used urethane (1.5 g/kg, Sigma-Aldrich, St. Louis, MO, USA) to deeply anesthetize the animals and we performed transcardially perfusion with 0.9% saline solution with sodium nitrite (1 g/l solution) and heparin (200 μl of heparin 25,000 UI/l of solution) following a solution of 4% paraformaldehyde (PFA; pH 7.4; Sigma-Aldrich, St. Louis, MO, USA). After dissection technique, the muscles were post- fixed in 4% paraformaldehyde during 2 h and then, cryoprotected (30% sucrose solution). Freezing of the muscles was carried out in isopentane (−40 °C, Sigma-Aldrich, St. Louis, MO, USA) and they were stored at −80 °C until the performance of coronal sections (25 μm) in a freezing microtome (Leica, model CM1850). For all the other techniques (plasma, oxidative metabolism, capillary density and electronic microscopy), rats were euthanized by conscious decapitation 24 hours after the behavioral test, and animal’s blood was collected to dosage of plasma corticosterone. Then, their heads were divided sagittally, and the right and left MPMs were dissected.

### Plasma corticosterone assay

This experiment was carried out to determine the effectiveness of protocols in reproducing stress-like hormonal alterations. Corticosterone plasma concentrations, a stress index, were measured using Luminex method (Milliplex), an immunoassay, that allows the identification of microspheres (bioassays on the surface of fluorescence-encoded plastic beads) which a flow analyzer can read through two lasers and digital signal processors.

### Oxidative metabolism analysis

The detection of Succinate Dehydrogenase was used to analyses metabolic activity of MPM fibers in different experimental conditions. Both, right and left MPMs, were mounted in Tissue-tekR, O.C.T. (optimal cutting temperature) solution, and then, they were frozen in isopentane cooled in liquid nitrogen (−150 °C). The specimens were kept at −80 °C until the use. Cross-sections of 10 μm thick were cut at −20 °C using a Leica cryostat microtome. The histochemical reaction for succinate dehydrogenase in these sections, demonstrated the muscle metabolic pattern^30^. The determination of the metabolic activity of the different fiber types followed the criteria established by Peter *et al*.^32^. It was considered the dark fibers as the most oxidative metabolic activity. The same examiner was responsible for quantifying the test, which prevents a varied tone interpretation of the fibers. The muscle sections were photographed using a photomicroscope (Leica MZ125 connected to a digital camera, Germany). A test-system composed of 90 points was used to quantity the images, according to Weibel *et al*.^33^. Image J software (Free software, http://rsb.info.nih.gov/ij) was used to count the points.

### Assessment of capillary density

We identified capillaries by immuno-histochemical analysis for Laminin proteins that is a component in the basement membrane of capillaries. The fluorescence method was used to the reaction, that was performed in MPM sections of 5 μm of thickness. They were immersed in a solution of BSA (3%, Pentex, USA) and they were washed three times with PBS, then, the slides were incubated overnight with the primary antibodies: Anti-Laminin antibody (1:1000, ab11575). After this period, sections were washed three times again in PBS and they were subjected to amplification reaction using the biotinylated secondary antibody (anti-mouse, Kit LSAB – HRP, DAKO Cytomation, Denmark). For each animal, it was captured five images, randomly, with 40× magnification, by a camera attached to a ZEISS microscope (ZEISS AxioImager. Z2, Germany).

### Ultrastructural analysis

For Transmission Electron Microscopy (TEM) analysis, we excised the medial pterygoid muscles, cutting them to fragments of 1 mm. These fragments were immersed in a sodium phosphate buffer 0.1 M containing 2.5% glutaraldehyde and 2% paraformaldehyde (modified Karnovsky solution). Osmium tetroxide solution at 4 °C was used to post-fixation and then, the fragments were immersed in 5% uranyl acetate aqueous during 24 hours. A dehydratetion of the tissues was made with ascending series of alcohol and they were immersed in propylene oxide. The end process was an infiltration in a mixture of pure resin and propylene oxide (1:1) and then, embedded in Spurr resin until complete polymerization. The tissues in resin were sectioned in a thickness of 55 nm. They were collected on 200 mesh networks, contrasted with uranyl acetate. The sections were examined by a JEOL 1010 (Japan) transmission electron microscope.

### Statistical analysis

Data are expressed as mean ± SD. Two-way ANOVA was employed to evaluate the effects of chronic unpredictable stress and exodontia. Post hoc analysis was conducted using Student-Newman-Keuls. The level of significance was set at *P* < 0.05.

### Ethics approval

The animal experimental protocol was approved by the Animal Use and Ethics Committee of Ribeirão Preto (certificate number: 12.1.418.53.0), all efforts were made to decrease the number of rats used and to minimize animal suffering.

## Data Availability

All relevant data of this research are within the manuscript.
